# Emotional Reactivity and Internalizing Symptoms in Middle Childhood: Integrating Autonomic and Behavioral Markers of Social Fear and Positive Affect

**DOI:** 10.1002/dev.70056

**Published:** 2025-06-17

**Authors:** Madison Politte‐Corn, Rebecca J. Brooker, H. Hill Goldsmith, Kristin A. Buss

**Affiliations:** ^1^ Department of Psychology The Pennsylvania State University University Park Pennsylvania USA; ^2^ Department of Psychological and Brain Sciences Texas A&M University College Station Texas USA; ^3^ Department of Psychology University of Wisconsin–Madison Madison Wisconsin USA

**Keywords:** childhood, fear, internalizing, PEP, positive affect, RSA

## Abstract

Emotional reactivity is a well‐validated corollary of children's risk for internalizing psychopathology and can be indexed by autonomic and behavioral measures. Yet, it is unclear whether and how autonomic and behavioral markers of emotional reactivity interact to characterize internalizing symptoms and whether these associations differ based on emotional context. As such, the current study aimed to (1) clarify associations between autonomic (RSA, PEP) and behavioral measures of emotional reactivity across two tasks designed to elicit fear and positive affect in social contexts and (2) examine the unique and combined associations between autonomic and behavioral reactivity during these tasks and internalizing symptoms. Participants were 328 children aged 6–10 (*M* = 7.91, *SD* = 0.97; 50% female; 94% White). Behavioral displays of positive affect during a parent task were associated with RSA withdrawal, but there were no significant associations between autonomic reactivity and behavioral displays of stranger fear. RSA augmentation during the parent task was associated with lower internalizing symptoms at average or high levels of positive affect. Finally, higher stranger fear was associated with higher internalizing symptoms only when coupled with reciprocal parasympathetic activation. These findings suggest context‐specific patterns of autonomic activation that are differentially associated with internalizing symptoms.

## 1 Introduction

Emotional reactivity is a well‐validated corollary of children's risk for internalizing psychopathology and can be measured across levels of analysis, including peripheral physiology (e.g., the autonomic nervous system) and behavior (T. Beauchaine 2001; Deutz et al. 2019; Gabel et al. 2023; McClelland and Cameron 2012; Nigg 2006). Yet, it is unclear whether and how autonomic and behavioral markers of emotional reactivity interact to characterize internalizing symptoms. Moreover, these associations may differ based on the type of emotion elicited (Fortunato et al. 2013; Fry et al. 2022; Gatzke‐Kopp and Ram 2018; Hinnant and El‐Sheikh 2009; Quiñones‐Camacho and Davis 2019). Understanding this variation across contexts allows us to better characterize and predict internalizing problems. As such, the current study aimed to address this gap by examining associations between autonomic and behavioral reactivity and the early emergence of internalizing symptoms across tasks designed to elicit fear and positive affect, two temperamental traits central to etiological models of internalizing psychopathology (Fox and Pine 2012; Hettema et al. 2020; Morales et al. 2022).

### 1.1 Associations Between Autonomic and Behavioral Measures of Emotional Reactivity

Within the autonomic nervous system, *respiratory sinus arrhythmia* (RSA; also referred to as vagal tone) and *pre‐ejection period* (PEP) are two measures that reliably index changes in parasympathetic and sympathetic responses, respectively (Berntson et al. 1994). RSA is an index of heart rate variability that measures changes in heart periods (*r* to *r* intervals) at the frequency of respiration (Berntson et al. 1993; Holzman and Bridgett 2017). PEP measures the time in milliseconds between the onset of ventricular depolarization and the opening of the semilunar valve in the heart, indicating the onset of the left ventricular ejection (Cacioppo et al. 1994). These autonomic measures can be leveraged as physiological markers of emotional reactivity when measured in the context of an environmental stressor or challenge (Graziano and Derefinko 2013; Porges 1995).

Clarifying associations between these autonomic indices of emotional reactivity and behavior is an important precursor to understanding how these domains may interact to characterize internalizing psychopathology. One theory is that fluctuations in RSA reflect undifferentiated emotional arousal and sufficiently facilitate the necessary behavioral response without changes in PEP (Brosschot et al. 2016; Brosschot et al. 2017; Porges 1995, 2007). Consistent with this interpretation, both toddlers and children show RSA withdrawal without showing changes in PEP during various emotional stressors (Buss et al. 2005; Talge et al. 2008). However, it is important to note that the Polyvagal perspective suggests distinct functions of the parasympathetic nervous system in threatening versus affiliative social contexts, consistent with the contention that social behavior is at the core of autonomic development (Porges 2007). Specifically, while parasympathetic withdrawal is thought to facilitate behavioral responses to social threat, parasympathetic augmentation is thought to facilitate social affiliation (Porges 1995, 2007). Thus, there may be distinct patterns of autonomic reactivity and relations with internalizing risk depending on the social context in which autonomic function is measured. Other theoretical perspectives have posited similar emotion‐specific patterns of cardiovascular reactivity in which positive and negative emotions have unique autonomic correlates (Fry et al. 2022; Levenson et al. 1990).

Colloquial knowledge portrays fear physiology as comprising increases in heart rate and peripheral physiology that are served by greater sympathetic activity and less parasympathetic control. This is consistent with empirical studies indicating a positive association between RSA withdrawal and behavioral displays of fear in children (Quiñones‐Camacho and Davis 2019; Viana et al. 2021). Similarly, a shorter PEP, indexing sympathetic augmentation, was associated with more fear behavior during a fear‐eliciting task in toddlers (Buss et al. 2004). However, some studies find the opposite pattern, such that greater RSA was associated with more displays of fear in early childhood across a variety of fear‐eliciting tasks, potentially reflective of physiological dysregulation (Buss et al. 2018; Calkins and Dedmon 2000; Talge et al. 2008). Less research has examined autonomic reactivity within positive‐affect‐eliciting tasks, though preliminary evidence indicates a positive association between sympathetic augmentation or parasympathetic withdrawal and displays of positive affect in both infants (Field et al. 1995; Stifter et al. 1989) and adolescents (Siciliano et al. 2023). Also, joint changes in RSA and PEP may more reliably differentiate behavioral arousal compared to singular measures. Indeed, one recent study of kindergarteners found that approach‐oriented emotions such as anger and happiness were typically characterized by reciprocal coordination across autonomic branches, whereas avoidance‐oriented emotions such as fear and sadness were characterized by a lack of autonomic coordination (Fry et al. 2022). Taken together, existing literature includes mixed findings regarding associations between fear and autonomic reactivity, as well as a paucity of research on autonomic correlates of positive affect and interactions between autonomic branches in characterizing behavioral arousal.

### 1.2 Autonomic Reactivity as a Predictor of Internalizing Psychopathology

It remains unclear whether RSA withdrawal or augmentation in response to an environmental challenge is associated with better socioemotional outcomes (T. Beauchaine 2001; Graziano and Derefinko 2013; Hastings et al. 2008; Holzman and Bridgett 2017; Obradović and Finch 2017). During a task in which children tell a story in front of two experimenters, gradual RSA withdrawal was associated with better executive function (Obradović and Finch 2017). Further, meta‐analytic evidence indicates that, across a variety of cognitive, social, and emotional tasks, greater levels of RSA withdrawal are related to fewer internalizing, externalizing, and academic problems in children (Graziano and Derefinko 2013). These studies suggest that RSA withdrawal in response to an environmental challenge is adaptive, and failure to withdraw parasympathetic resources in response to a challenge may be associated with psychopathology risk (Graziano and Derefinko 2013; Obradović and Finch 2017). Notably, these findings are consistent with previously mentioned studies reporting positive associations between RSA and fear (Buss et al. 2018; Calkins and Dedmon 2000; Talge et al. 2008). However, other studies have found the opposite pattern, such that pronounced vagal withdrawal in response to emotional challenges, indicating increased arousal, is associated with greater emotional dysfunction (T. P. Beauchaine 2015; T. P. Beauchaine et al. 2019). For instance, a few studies with early and middle childhood samples have reported that decreased RSA during social threat is positively associated with concurrent and longitudinal shyness (Hassan and Schmidt 2024; Poole and Schmidt 2021) and longitudinal internalizing symptoms (Hinnant and El‐Sheikh 2009). Consistent with this, other studies suggest that RSA augmentation, rather than withdrawal, is associated with better behavioral self‐regulation and fewer internalizing problems in children (Hastings et al. 2008; Holzman and Bridgett 2017). One likely reason for these inconsistent findings is that the association between RSA activation and socioemotional well‐being depends on the specific task demands or emotional context (Fortunato et al. 2013; Fry et al. 2022; Gatzke‐Kopp and Ram 2018; Hinnant and El‐Sheikh 2009; Quiñones‐Camacho and Davis 2019).

Less work has examined the association between PEP reactivity and internalizing symptoms. One study found that PEP lengthening, consistent with less sympathetic arousal, during a negative mood induction was associated with higher attentional control and internalizing symptoms (Andersen et al. 2020). Consistent with theoretical perspectives positing that parasympathetic withdrawal initiates the normative cardiovascular response to stress (Brosschot et al. 2016; Brosschot et al. 2017; Porges 1995, 2007), joint changes in PEP and RSA might reflect a more adaptive autonomic response than changes in PEP alone. Indeed, one study found that children who exhibited PEP shortening without RSA change across a series of cognitive and emotional challenges demonstrated poorer executive functioning compared with children who exhibited PEP shortening in conjunction with RSA withdrawal (Zeytinoglu et al. 2022).

Importantly, the association between autonomic reactivity and internalizing risk likely depends on the emotional context in which autonomic function is measured (Fortunato et al. 2013; Fry et al. 2022; Gatzke‐Kopp and Ram 2018; Hinnant and El‐Sheikh 2009; Quiñones‐Camacho and Davis 2019). For instance, parasympathetic regulation across various cognitive and emotion‐eliciting tasks differentially predicts children's trait emotional reactivity and regulation (Quiñones‐Camacho and Davis 2019). Further, coordination between autonomic branches differentially relates to longitudinal autonomic development when it occurs in the context of approach‐oriented emotions (e.g., happy) versus avoidance‐oriented emotions (e.g., fear; Fry et al. 2022). As such, examining reactivity across multiple emotional tasks is critical to understanding the autonomic correlates of internalizing symptoms.

### 1.3 Fear and Positive Affect as Predictors of Internalizing Psychopathology

Etiological models of internalizing psychopathology underscore high fearfulness and low positive affect as two temperamental traits particularly indicative of risk (Fox and Pine 2012; Hettema et al. 2020; Klein and Mumper 2018; Kujawa et al. 2020; Morales et al. 2022). Myriad empirical studies have shown that heightened fearful behavior predicts later internalizing problems, particularly social anxiety (e.g., Biederman et al. 2001; Buss 2011; Chronis‐Tuscano et al. 2009; Hirshfeld‐Becker et al. 2007; Kagan et al. 1999; Morales et al. 2015). For instance, Kagan et al. (1999) demonstrated that approximately 46% of infants who exhibited heightened fearful behavior in response to novelty showed anxious symptomatology at age 7, and later work has shown that this association between early temperamental fear and internalizing symptomatology persists into adolescence (Chronis‐Tuscano et al. 2009; Biederman et al. 2001; Goldsmith et al. 2022; Hirshfeld‐Becker et al. 2007) and adulthood (Tang et al. 2020). Importantly, the cumulative evidence suggests that fearful temperament is a predisposing or vulnerability factor for internalizing rather than a precursor (Klein and Mumper 2018), meaning that the presence of other neurobiological risk factors is important in determining which children will develop internalizing problems. Specifically, fearful children whose physiological regulatory systems amplify rather than attenuate fear may be particularly at risk (Sylvester and Pine 2018).

Compelling evidence indicates that reduced positive affect is a key predictor of internalizing psychopathology (Hankin et al. 2017; Khazanov and Ruscio 2016; Morales et al. 2022; Watson et al. 2005). For example, one study demonstrated that observed positive affect in infancy negatively predicted internalizing symptoms, but not general psychopathology, across ages 7–12 (Morales et al. 2022). Others have shown this unique association between low positive affect and internalizing risk in two independent samples of children and adolescents (Hankin et al. 2017). Thus, behavioral and physiological reactivity associated with fear and positive affect are particularly important to characterizing internalizing risk in youth.

### 1.4 The Present Study

Despite well‐documented, though mixed, associations between autonomic and behavioral indices of emotional reactivity and internalizing symptoms, whether and how these domains interact to predict symptomatology is not well understood. The first aim of the present study was to clarify associations between cardiovascular and behavioral measures of emotional reactivity, using two tasks designed to elicit social wariness in the presence of a stranger and positive affect in the presence of a caregiver, respectively. Tasks that were social in nature were selected for analysis, consistent with polyvagal perspectives that position social behavior at the core of autonomic development (Porges 1995, 2009, 2021). Our approach allowed us to test competing hypotheses. First, higher behavioral reactivity may be associated with lower RSA, but not PEP, relative to baseline, reflecting theoretical positions that fluctuations in RSA reflect undifferentiated emotional arousal and sufficiently facilitate the necessary behavioral response without changes in PEP (Brosschot et al. 2016; Brosschot et al. 2017; Porges 1995, 2007). An alternate hypothesis, though less represented in the broader developmental literature, is that distinct autonomic patterns would emerge across tasks, such that RSA withdrawal would be observed during the fear‐eliciting task but RSA augmentation would be observed during the positive affect task (Porges 2007).

Our second aim was to examine the unique and combined associations between autonomic and behavioral reactivity and internalizing symptoms. We focused on the emotions of fear and positive affect, given considerable extant literature linking these temperamental traits to later internalizing psychopathology (e.g., Buss 2011; Chronis‐Tuscano et al. 2009; Kelsey et al. 2023; Morales et al. 2020). For this aim, we hypothesized that greater behavioral displays of fear during the stranger task and lower behavioral displays of positive affect during the parent task would be associated with higher internalizing symptoms, consistent with prior research (Buss 2011; Chronis‐Tuscano et al. 2009; Hankin et al. 2017; Morales et al. 2020). Drawing from Polyvagal theory (Porges 1995, 2007, 2009), we further hypothesized distinct associations between the autonomic measures and internalizing symptoms across tasks. During the dyadic positive affect task, we expected that the adaptive response would be RSA augmentation and that decreases in RSA or sympathetic augmentation would be associated with higher internalizing symptoms, consistent with polyvagal models of social affiliation (Porges 2009, 2021). As the stranger task is putatively low threat but still requires behavioral engagement, we expected that the adaptive response would be RSA withdrawal, and parasympathetic or sympathetic augmentation would be associated with higher internalizing symptoms. Finally, we hypothesized interactions between autonomic and behavioral indices of emotional reactivity in the prediction of internalizing symptoms, given heterogeneity in these main effects (Graziano and Derefinko 2013; Hinnant and El‐Sheikh 2009; Klein and Mumper 2018; Morales et al. 2022). Prior work has suggested that approach‐oriented emotions, such as positive affect, should be associated with reciprocal coordination across autonomic branches, whereas avoidance‐oriented emotions, such as fear, should be associated with uncoupled autonomic activity to simultaneously facilitate vigilance and focused attention (Fry et al. 2022; Gatzke‐Kopp and Ram 2018). Consequently, we expected that deviation from these normative patterns of activation would be associated with internalizing symptoms.

We examined these questions in a middle childhood sample to clarify these associations prior to the peripubertal period and inform subsequent developmental trajectories, given that puberty is characterized by substantial neurobiological and social changes and increased risk for clinical levels of internalizing symptoms (Nelson et al. 2016; Pfeifer and Allen 2021). Further, parental socialization of emotion is well underway by middle childhood, allowing for more individual differences in emotional reactivity and regulation compared to earlier in development (Eisenberg et al. 1998; Lunkenheimer et al. 2020).

## 2 Method

### 2.1 Participants

Participants included 328 twins (163 girls; 83 monozygotic pairs; 58 same‐sex dizygotic pairs; 23 opposite‐sex dizygotic pairs) between the ages of 6 and 10 (*M* = 7.91, *SD* = 0.97) who participated in a larger twin study examining temperament (Affect, Psychophysiology, and Heritability Encoded by the Conduct of Twins). The study employed a twin design to allow examination of genetic factors, which are not the subject of the current study. Recruited through birth records, participating children were above the 10th percentile for twin‐specific birth weight and showed no significant birth complications or congenital anomalies. Based on parental reports of child ethnicity, the sample was predominantly White (94%), with 1.5% of parents identifying their children as Black/African American and 4.5% identifying their children as multiracial. Families were primarily middle‐class (Hollingshead Index score *M* = 45.80, *SD* = 10.93) and lived in two‐parent homes, which mirrored the local population.

### 2.2 Procedure

As part of a larger study, families completed a 2.5‐h laboratory visit for psychophysiological data collection. After families provided consent and assent, each child provided saliva and DNA samples. An experimenter then placed sensors on the child to record cardiovascular activity, brain electrical activity, and eye blink responses. Only cardiovascular data are included in this study. Except for one task, the child was either alone in the testing room or with an experimenter. Each twin was then taken to separate testing rooms where the child participated in a series of five challenging tasks that ranged in length from 1 to 8 min (modified from the Lab‐TAB protocol; Gagne et al. 2011; Goldsmith et al. 1994). Tasks were completed in a standard order, and cardiovascular data were collected during all tasks. Only *Pop‐Out Toy* and *Stranger Conversation* are included in this study because they were designed to elicit positive affect and social fear, respectively, both of which are associated with internalizing risk (Buss 2011; Buss et al. 2020; Chronis‐Tuscano et al. 2009; Kelsey et al. 2023; Morales et al. 2020). After the last task, the sensors for physiological monitoring were removed, and the experimenter collected a final saliva sample. All episodes were videotaped through a two‐way mirror. The Institutional Review Board at the University of Wisconsin–Madison approved this study. Parents of children provided informed consent, and all children provided informed assent.

#### 2.2.1 Behavioral Tasks

##### 2.2.1.1 Fear‐Eliciting Task

The *Stranger Conversation* task elicits social fear and social wariness during middle childhood (Talge et al. 2008). In this task, a stranger entered the room and engaged the child in a conversation about their hobbies and interests. If the child spoke, the stranger gave two additional prompts before thanking the child and starting the next part of the task. If the child did not speak, the stranger waited 90 s and repeated the initial prompt. If the child remained silent, the stranger waited an additional 90 s before making a final prompt. The task lasted an average of 107.87 s (*SD* = 57.61 s).^1^

##### 2.2.1.2 Dyadic Positive Affect Task

The *Pop‐Out Toy* task elicits high‐intensity pleasure/positive affect during an interaction with a caregiver in middle childhood (Pfeifer et al. 2002). In this task, the child was sitting in a chair at a table with an experimenter sitting adjacent to them. The experimenter gave the child a can that appeared to contain nuts but contained a slinky toy snake. The experimenter asked the child to help open the can and then opened the lid and allowed the slinky toy to pop out of the can. In a conspiratorial voice, the experimenter suggested that the child surprise their parent by pretending that the second can was also filled with nuts. The experimenter instructed the child to remain seated and wait to surprise them until their parent sat down next to them, then left the room and returned after giving the child the opportunity to surprise their parent. This study used only data from the portion of the task in which the child surprised their parent, which is divided into five 10‐s epochs beginning when the parent opens the can. Therefore, the duration of this portion of the task was consistent across participants and lasted approximately 50 s.

#### 2.2.2 Cardiovascular Data Collection

Band sensors were placed on the child to collect impedance cardiography (ZCG) and electrocardiography (ECG). These bands were placed around the child's neck, below their arms, and around their torso at the base of the ribcage. Spot sensors were placed on the child's clavicle bones and torso, just below the right underarm. After a 4‐minute pre‐task baseline in which the child was instructed to remain relaxed, not to talk, and to sit still, cardiovascular data were collected during the behavioral tasks described above and during a 4‐minute post‐task baseline at the end of the study.

A noninvasive, CIC‐1000 Cardiac Output Monitor was used to collect ZCG data. The ZCG signal measured volumetric changes in the heart and systolic time intervals, including pre‐ejection time and left ventricular ejection time. A 500‐µA, 40‐kHz oscillating current (both harmless and undetectable) was passed through the two outer current electrodes. Separate channels recorded ECG, basal thoracic impedance (Zo), change in impedance (DZ), and the first derivative of the change in thoracic impedance during systole (dZ‐dt) from the two inner recording band electrodes.  The raw, continuous ECG signal was extracted from the SORBA system and separately pushed through a bandpass filter at 30 and 100 Hz, amplified 20 K, and sampled at 500 ms, following the methods of Buss et al. (2004, 2005).

### 2.3 Measures

#### 2.3.1 Coding of Displayed Emotion

For each task, behavioral coding was completed by undergraduate students who achieved reliability with a graduate‐level master coder (Kappas >0.70 for at least 10 participants). The master coder double‐coded approximately 20% of the sample. Kappas for all variables ranged from 0.69 to 0.89 (*M* = 0.77). All tasks were scored in 10‐s epochs. The maximum intensity of facial emotions was scored using the AFFEX definitions (Izard et al. 1983). Following this coding scheme, the reliable coders examined movement in three regions of the face: brow, eye/nose/cheek, and mouth. The intensity of emotions was rated as follows: 0 = *no codable movement in facial regions*, 1 = *one facial region showed movement*, 2 = *two facial regions showed movement or definite expression in one region* (e.g., eyes), and 3 = *a change in all 3 facial regions or strong impression of the emotion*. The intensity of bodily fear, avoidance, and vigor of enthusiasm was scored using the following intensity scale: 0 = *no detectable bodily display of emotion/behavior*, 1 = *low bodily display of emotion/behavior*, 2 = *moderate bodily display of emotion/behavior*, and 3 = *high bodily display of emotion/behavior*. Definitions of these codes are provided within the task descriptions below.

##### 2.3.1.1 Stranger Conversation

One team of reliable coders rated behaviors associated with fear and wariness. Facial fear was characterized by partially raised, straight brows that were drawn in; widened eyes showing more white than usual; a raised upper eyelid; and a slightly opened mouth with the corners pulled straight back. Bodily fear was coded when children showed decreased motor activity, bodily tension, startling, or freezing. Avoidance behaviors reflected children's efforts to maintain or increase distance from the stranger (e.g., turning body away, covering face, crossing arms, sinking in seat). Dichotomous codes (0 = *absence of behavior*, 1 = *presence of behavior*) were used to rate the following behaviors: nervous fidgeting, whispered speech, and verbal disfluencies. The latencies (in seconds) from the start of the task until the child spoke, displayed a fear response, or displayed fidgeting behaviors were also recorded. The latency to show a fear response and the latency to show fidgeting were reversed so that smaller numbers indicated a faster onset of fear or fidgeting behaviors.

##### 2.3.1.2 Pop‐Out Toy

A second team of reliable coders rated behaviors indicative of positive affect. Using the intensity scale described above, intensity of smiling was characterized by raised cheeks, corners of the mouth pulled back and up, and a furrow between the eyebrows. Vigor of enthusiasm was operationalized as interest in the game, approach behaviors, animation, and positive motor activity (e.g., kicking legs excitedly). Dichotomous codes (0 = *absence of behavior*, 1 = *presence of behavior*) were used to rate laughter. The peak intensity of positive vocalizations was rated on a 4‐point scale, such that 0 reflected a lack of positive vocalizations, and a score of 3 represented extremely positive vocalizations.

#### 2.3.2 Cardiovascular Scoring

##### 2.3.2.1 PEP

PEP was used to index SNS‐influenced cardiovascular activity. PEP was calculated as the time in milliseconds from the beginning of the electrical systole (corresponding to the Q wave of the ECG) to the beginning of the mechanical contraction (corresponding to the peak of the R spike on the ECG). PEP was calculated offline using CIC‐1000 Impedance Cardiograph software, Version 7.2 (SORBA Medical Systems 1997). For both tasks, PEP was calculated in 30‐s epochs. Algorithms in the CIC‐1000 software automatically marked the dZ/dt B point and the beginning of the QRS complex on the ECG waveform. The children's height, weight, and distance in inches between the two inner band electrodes were entered to accurately calculate PEP. For each epoch, the software calculated a PEP score, or the time between the onset of the Q wave in the ECG waveform and the B point of the dZ/dt waveform. The software also flagged abnormalities in the data that were later examined and edited when necessary, and PEP was recalculated. Across both tasks, 284 children provided usable PEP data.

##### 2.3.2.2 RSA

RSA measured PNS‐influenced cardiovascular activity. To calculate RSA, the raw ECG wave was extracted from the SORBA system, filtered, and transformed into files containing interbeat intervals (IBIs), or the time between heartbeats, using a program that had adjustable thresholds to detect R waves. The IBI files were entered into MXedit software, and the data were cleaned to identify and edit artifacts. The IBIs were subjected to a moving polynomial filter (with band‐pass set to 0.24–1.04 Hz, the frequency of spontaneous respiration in children), and RSA was computed as the natural logarithm of the variance on 30‐s epochs (Porges and Bohrer 1990). Across both tasks, 284 children provided usable RSA data.

#### 2.3.3 Child Internalizing Symptoms

Children's internalizing symptoms were measured via maternal and paternal reports on the MacArthur Health Behavior Questionnaire (HBQ; Armstrong and Goldstein 2003). The HBQ is a 140‐item scale that assesses behaviors consistent with internalizing and externalizing symptoms in middle childhood. Parents rated the extent to which items such as “Worries about doing better at things” describe their child on a 3‐point scale (0 = *rarely applies*, 1 = *applies somewhat*, and 2 = *certainly applies*). For the current analyses, we focus on the *Internalizing Tendencies* scale consisting of the Depression, Separation Anxiety, and Overanxious subscales. HBQ scales show high internal reliability; for the present study, Cronbach's alphas for scale scores ranged from 0.83 to 0.89 for paternal reports and from 0.83 to 0.88 for maternal reports. Prior research has supported the general validity and clinical utility of the HBQ for assessing psychopathology in young children (Luby et al. 2002). Paired samples *t*‐tests indicated that maternal and paternal reports of internalizing symptoms did not significantly differ in the current sample (*p* > 0.70). Further, maternal and paternal reports of internalizing symptoms were modestly correlated (*r* = 0.48, *p* < 0.001). Consequently, we averaged maternal and paternal reports of internalizing symptoms in the main analyses but also report the results of sensitivity analyses testing the two informants separately. Based on established clinical cutoff scores for the HBQ subscales (Luby et al. 2002) and combined maternal and paternal reports, one participant (0.3%) met the clinical cutoff for depression (mean score >0.85), 26 (7.9%) met the clinical cutoff for separation anxiety (mean score >0.65), and five (1.5%) met the clinical cutoff for generalized anxiety (mean score >0.90).

### 2.4 Data Reduction

#### 2.4.1 Behavioral Data Reduction

Following Lab‐TAB coding procedures (Gagne et al. 2011; Goldsmith et al. 1994), coded behaviors were averaged across epochs within each emotion‐eliciting task. These variables were standardized using a *z*‐transformation and then averaged to create fear and positive affect composite scores. Specifically, the stranger fear composite score was composed of avoidance (Cronbach's α = 0.93 across epochs), bodily fear (Cronbach's α = 0.92 across epochs), negativity (Cronbach's α = 0.92 across epochs), and latency to first fear response in seconds (reversed). The positive affect composite score comprised intensity of smiling (Cronbach's α = 0.82 across epochs), enthusiasm (Cronbach's α = 0.82 across epochs), laughter (Cronbach's α = 0.68 across epochs), and positive vocalizations (Cronbach's α = 0.60 across epochs). Principal components analysis confirmed that these individual behaviors loaded onto a single factor within each task, with factor loadings ranging from 0.65 to 0.98. Descriptive statistics and bivariate correlations between these individual behavioral codes are presented in the Supporting Information.

#### 2.4.2 Cardiovascular Data Reduction

PEP and RSA reactivity during the emotion tasks were computed as unstandardized residual scores, partialing out the variance associated with the *pre‐task baseline* measures. Lower PEP reactivity scores indicate a faster PEP (SNS augmentation), and higher PEP reactivity scores indicate a slower PEP (SNS withdrawal). Negative RSA reactivity scores represent PNS withdrawal during the challenge tasks; positive RSA reactivity scores indicate PNS augmentation. After winsorizing outliers (>3 *SD* from the mean; *n* = 3), the resulting distributions were relatively normal (skewness < 1).

### 2.5 Data Analysis

Because we were not interested in modeling random effects across twin pairs, cluster‐robust standard errors were used in all models to account for the clustered structure of our data. This approach treats the clustering mechanism (i.e., twins nested within families) as a nuisance, as opposed to multilevel modeling, which explicitly models the clustering mechanism and requires the researcher to make several statistical decisions and assumptions that, if incorrect, will bias both parameter estimates and estimates of their precision (McNeish and Stapleton 2016). Specifically, cluster‐robust standard errors account for dependencies within clusters that reduce the amount of unique information available in the sample size, reducing the total sample size used in the denominator when calculating standard errors (McNeish and Stapleton 2016).

Across all variables, 10.94% of cases were missing, and Little's MCAR test suggested that these data were not missing completely at random, *χ* ^2^ = 15.59, *p* = 0.008. Inspection of the missing data patterns revealed that children missing one cardiac variable were also likely to be missing the other, and such missingness was not significantly associated with any of the study variables. Full‐information maximum likelihood (FIML) was used to account for missing data in all regression models, as FIML outperforms other methods for handling missing data under the condition of MAR (Xiao and Bulut 2020). Data were analyzed using the *lavaan* package in R using maximum likelihood parameter estimates with robust standard errors (Rosseel 2012).

Prior to conducting the study analyses, we first examined whether sex, age, and socioeconomic status (Hollingshead index) should be included as covariates. Next, hierarchical linear regression models were computed to address the study aims. Specifically, for our first goal to examine associations between cardiovascular and behavioral measures of emotional reactivity, we entered stranger fear and positive affect as outcomes in separate models. Relevant covariates were entered at Step 1, residualized change in RSA and PEP from baseline (i.e., reactivity) were entered at Step 2, and an RSA × PEP interaction term was entered at Step 3 given evidence that joint changes in sympathetic and parasympathetic function more reliably predict behavior than singular autonomic measures (Fry et al. 2022). For our second goal to examine cardiovascular and behavioral measures of emotional reactivity as predictors of internalizing symptoms, we computed two additional sets of hierarchical regression analyses (examining each task separately) with internalizing symptoms entered as the outcome.^2^ Relevant covariates and the reactivity measures were entered at Step 1, two‐way interactions between the cardiovascular and behavioral measures were entered at Step 2, and a three‐way interaction between RSA, PEP, and behavioral reactivity was entered at Step 3. Interactions were probed at (*p* < 0.05) by examining simple slopes.

## 3 Results

### 3.1 Preliminary Analyses

Paired‐samples *t*‐tests indicated that mean RSA was significantly lower during the tasks relative to baseline [*t*(282) = 4.98, *p* < 0.001 for the positive affect task; *t*(272) = 13.76, *p* < 0.001 for the stranger task]. PEP was significantly faster during the stranger task relative to baseline [*t*(283) = 2.68, *p* = 0.008], but there were no significant mean‐level changes in PEP from baseline to the positive affect task [*t*(268) = 0.75, *p* = 0.45].

Child biological sex was associated with both PEP and level of internalizing symptoms; girls exhibited faster PEP at baseline than boys, *t*(326) = 2.00, *p* = 0.046, and higher PEP augmentation during the stranger task, *t*(326) = 2.17, *p* = 0.03. Girls also showed higher internalizing symptoms, *t*(326) = 2.29, *p* = 0.02. As Table 1 shows, older children showed significantly less stranger fear than younger children. Thus, we covaried for sex and age in later regression models predicting stranger fear and covaried for sex in models predicting positive affect and internalizing symptoms.

**TABLE 1:** Means, standard deviations, and correlations with confidence intervals.

Variable	*M*	*SD*	1	2	3	4	5	6	7
1. Age	7.91	0.97							
2. Positive affect	0.00	0.72	−0.11						
			[−0.21, 0.00]						
3. Stranger fear	0.00	0.50	−0.16^**^	0.26^**^					
			[−0.26, −0.05]	[0.15, 0.36]					
4. PEP reactivity: Pop‐out toy	0.00	0.01	−0.09	0.01	−0.04				
			[−0.21, 0.03]	[−0.11, 0.13]	[−0.16, 0.08]				
5. RSA reactivity: Pop‐out toy	0.00	0.64	0.04	−0.17^**^	0.01	0.26^**^			
			[−0.08, 0.15]	[−0.28, −0.05]	[−0.12, 0.13]	[0.14, 0.38]			
6. PEP reactivity: Stranger	0.00	0.01	0.06	0.01	−0.02	0.27^**^	0.02		
			[−0.06, 0.17]	[−0.11, 0.12]	[−0.14, 0.09]	[0.15, 0.38]	[−0.09, 0.13]		
7. RSA reactivity: Stranger	0.00	0.57	0.09	−0.12	−0.02	0.01	0.28^**^	0.15^*^	
			[−0.03, 0.21]	[−0.23, 0.00]	[−0.14, 0.10]	[−0.12, 0.14]	[0.16, 0.38]	[0.03, 0.27]	
8. Internalizing symptoms	0.29	0.17	0.03	0.18^**^	0.00	0.05	−0.20^**^	−0.01	−0.10
			[−0.08, 0.15]	[0.07, 0.28]	[−0.11, 0.12]	[−0.07, 0.17]	[−0.31, −0.08]	[−0.13, 0.11]	[−0.22, 0.02]

*Note:* Values in square brackets indicate the 95% confidence interval for each correlation, which is a plausible range of population correlations that could have caused the sample correlation.

*p < 0.05; **p < 0.01.

The Table 1 correlations show that the PEP reactivity measures were correlated across the two contexts of the pop‐out toy (eliciting positive affect) and the stranger conversation (eliciting fear) tasks. Within both the pop‐out toy and the stranger conversation contexts, PEP reactivity and RSA reactivity were positively correlated, indicating overall reciprocal autonomic coordination. Across contexts, we note that RSA augmentation to the positive affect task was correlated with lengthened PEP in the stranger conversation task. Only two significant associations occurred between cardiovascular reactivity measures and behavior, and both involved RSA reactivity to the positive affect task. Both positive affect and internalizing symptoms were negatively correlated with RSA augmentation in the pop‐out toy episode. Stranger fear was not significantly associated with any of the cardiovascular measures or internalizing symptoms. Unexpectedly, behavioral displays of positive affect were positively correlated with stranger fear and internalizing symptoms.

### 3.2 Aim 1: Associations Between Cardiovascular and Behavioral Reactivity

Results of the regression analyses predicting behavioral displays of emotion are presented in Table 2. Findings were consistent with overall emotion‐specific autonomic patterns, such that positive affect was associated with decreased RSA reactivity to a positive stimulus, reflecting RSA withdrawal (*β* = −0.19, cluster‐robust *SE* = 0.07, *p* = 0.01), but this pattern of RSA withdrawal was not observed during the stranger fear episode (*β* = 0.01, cluster‐robust *SE* = 0.06, *p* = 0.94). There were no significant main effects of PEP reactivity or interactions between RSA and PEP reactivity in predicting positive affect or stranger fear (*p*s > 0.05).

**TABLE 2:** Regression analyses predicting behavioral displays of positive affect and stranger fear.

Outcome: Positive affect		*β* (cluster‐robust SE)	*z*‐value	*p*
Step 1	Sex	0.30 (0.13)	2.36	0.02
Step 2	PEP reactivity	0.05 (0.07)	0.70	0.48
	RSA reactivity	−0.20 (0.07)	−2.67	0.01
Step 3	RSA × PEP reactivity	0.02 (0.07)	0.31	0.76

Outcome: Stranger fear		*β* (cluster‐robust SE)	*z*‐value	*p*
Step 1	Age	−0.15 (0.06)	−2.35	0.02
	Sex	0.26 (0.11)	2.27	0.02
Step 2	PEP reactivity	−0.02 (0.07)	−0.28	0.78
	RSA reactivity	0.01 (0.06)	0.08	0.94
Step 3	RSA × PEP reactivity	−0.02 (0.07)	−0.27	0.79

### 3.3 Aim 2: Cardiovascular and Behavioral Measures of Emotional Reactivity as Predictors of Internalizing Symptoms

**FIGURE 1:**
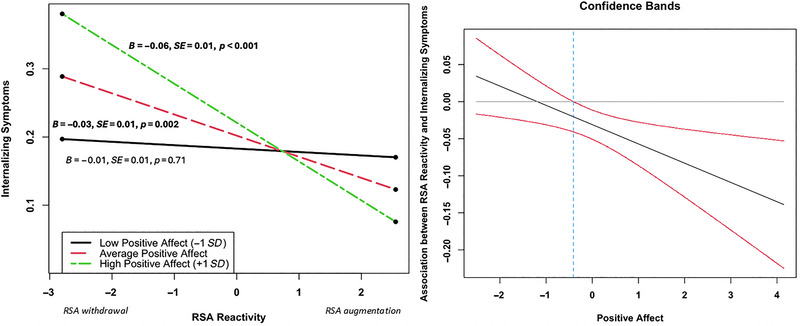
Two‐way interaction between RSA reactivity and behavioral displays of positive affect predicting internalizing symptoms.

Results of the regression analyses predicting internalizing symptoms are presented in Table 3. Consistent with hypotheses, there was a negative main effect of RSA reactivity during the positive affect task on internalizing symptoms (*β* = −0.03, *z* = −3.02, *p* = 0.003), indicating that RSA augmentation in this context was associated with lower internalizing symptoms. This effect was qualified by a significant two‐way interaction between RSA reactivity and behavioral displays of positive affect in predicting internalizing symptoms (*β* = −0.03, *z* = −2.24, *p* = 0.025). As seen in Figure 1, the negative association between RSA and internalizing symptoms was significant at average (simple slope = −0.03, *t* = −3.10, *p* = 0.002) and high (simple slope = −0.06, *t* = −3.83, *p* < 0.001) displays of positive affect but not when positive affect was low (simple slope = −0.01, *t* = −0.37, *p* = 0.71). The Johnson–Neyman region of significance indicated that the negative association between RSA reactivity and internalizing symptoms was significant at standardized positive affect scores above −0.41, to the right of the dashed line in Figure 1. Unexpectedly, there were no main effects of positive affect or PEP on internalizing symptoms, nor was there a significant three‐way interaction between the behavioral and cardiovascular measures during the positive affect task predicting internalizing symptoms.

**TABLE 3:** Regression analyses of behavioral and cardiovascular reactivity to the pop‐out toy and stranger tasks predicting internalizing symptoms.

Task: Pop‐out toy		*β* (cluster‐robust SE)	z‐value	*p*
**Step 1**	Sex	0.05 (0.02)	2.31	0.02
	PEP reactivity	0.01 (0.01)	1.31	0.19
	RSA reactivity	−0.03 (0.01)	−3.02	0.003
	Positive affect	0.02 (0.01)	1.64	0.10
**Step 2**	RSA × PEP reactivity	0.01 (0.01)	0.86	0.39
	RSA reactivity × Positive affect	−0.03 (0.01)	−2.24	0.03
	PEP reactivity × Positive affect	0.02 (0.01)	1.47	0.14
**Step 3**	RSA × PEP reactivity × Positive affect	0.00 (0.01)	−0.17	0.87
**Task: Stranger conversation**		***β* (cluster‐robust SE)**	**z‐value**	***p***
**Step 1**	Sex	0.05 (0.02)	2.29	0.02
	PEP reactivity	0.00 (0.01)	0.03	0.98
	RSA reactivity	−0.02 (0.01)	−1.37	0.17
	Stranger fear	0.00 (0.01)	−0.36	0.72
**Step 2**	RSA × PEP reactivity	0.00 (0.02)	−0.21	0.83
	RSA reactivity × Fear	0.01 (0.01)	0.89	0.37
	PEP reactivity × Fear	0.01 (0.01)	1.07	0.29
**Step 3**	RSA × PEP reactivity × Fear	0.04 (0.02)	2.64	0.008

**FIGURE 2:**
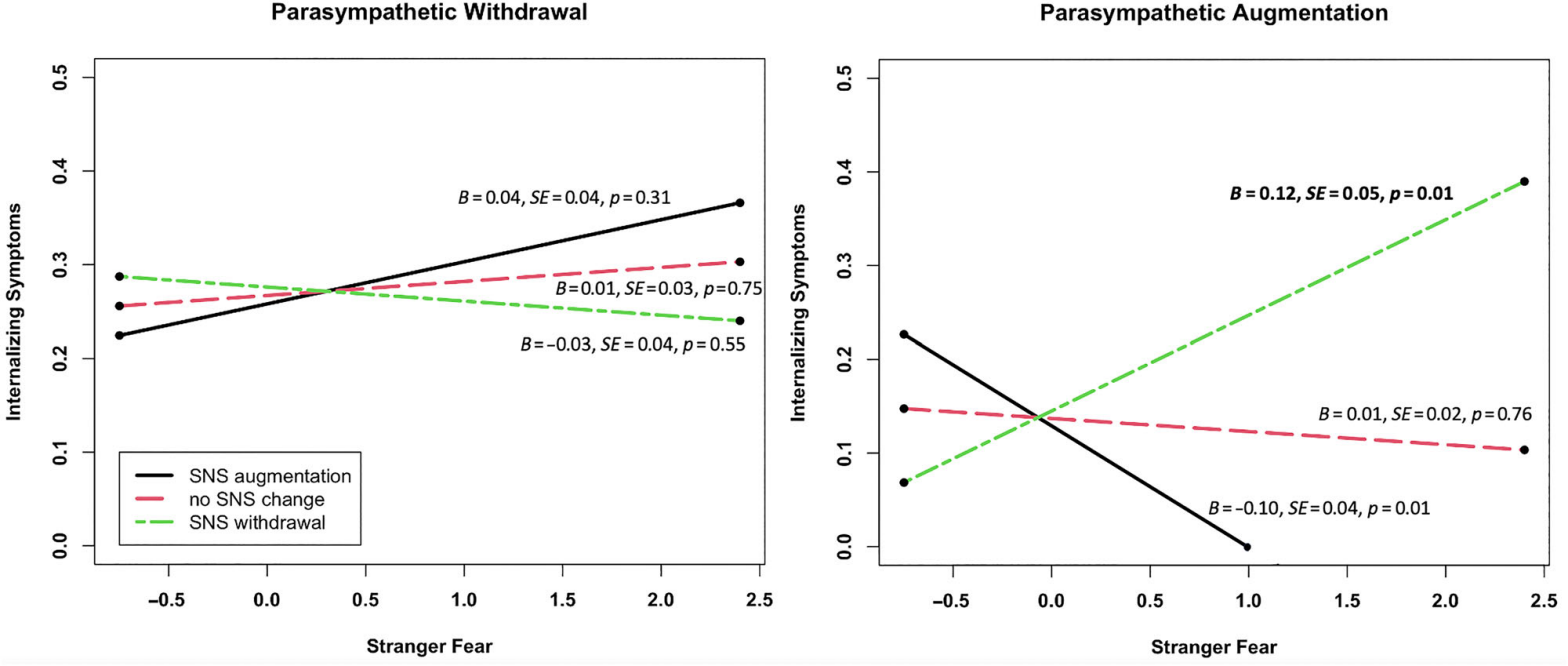
Three‐way interaction between RSA reactivity, PEP reactivity, and behavioral displays of stranger fear predicting internalizing symptoms.

Turning to reactivity during the fear task, we observed a significant three‐way interaction between RSA, PEP, and behavioral reactivity in the prediction of internalizing symptoms (*β* = 0.04, *z* = 2.63, *p* = 0.009). To probe this interaction, we split the data based on RSA withdrawal (unstandardized residual <0; *n* = 147) or RSA augmentation (unstandardized residual >0; *n* = 181) during the stranger task and tested the fear × PEP two‐way interactions for each group. As displayed in Figure 2, more fearful behavior was associated with higher internalizing symptoms only when coupled with reciprocal parasympathetic activation (i.e., RSA augmentation and PEP withdrawal; simple slope = 0.12, *t* = 2.56, *p* = 0.01), which was consistent with predictions. The simple slope between fear and internalizing symptoms was also statistically significant for children who exhibited parasympathetic and sympathetic co‐activation (simple slope = −0.10, *t* = −2.73, *p* = 0.01), although this effect fell within a restricted range of data and is therefore interpreted with caution. There were no significant main effects of RSA, PEP, or stranger fear on internalizing symptoms.

### 3.4 Sensitivity Analyses

Because maternal and paternal reports of internalizing symptoms were only modestly correlated (*r* = 0.48), we conducted sensitivity analyses for the main findings, testing the two informants separately. The negative association between RSA reactivity during the dyadic positive affect task and internalizing symptoms remained significant across informants (*β* = −0.03, *z* = −2.45, *p* = 0.014 for maternal report; *β* = −0.03, *z* = −2.17, *p* = 0.030 for paternal report). However, moderation of this effect by behavioral displays of positive affect was specific to father‐reported internalizing symptoms (*β* = −0.02, *z* = −1.23, *p* = 0.22 for maternal report; *β* = −0.03, *z* = −2.01, *p* = 0.044 for paternal report). Additionally, the significant three‐way interaction between RSA, PEP, and behavioral reactivity during the stranger task predicting internalizing symptoms was specific to mother‐reported internalizing symptoms (*β* = 0.06, *z* = 3.05, *p* = 0.002 for maternal report; *β* = 0.02, *z* = 1.14, *p* = 0.25 for paternal report). The pattern of the interaction effect for mother‐reported internalizing symptoms was consistent with that observed for combined maternal and paternal reports, such that fearful behavior was associated with higher internalizing symptoms only when coupled with reciprocal parasympathetic activation.

We also repeated the analyses testing the internalizing subscales separately, as fear behavior may be particularly relevant to anxiety development relative to depression. As expected, the three‐way interaction between RSA, PEP, and behavioral reactivity during the stranger fear task was significant for anxiety symptoms (*β* = 0.04, *z* = 2.45, *p* = 0.014), but not for depressive symptoms alone (*β* = 0.03, *z* = 1.62, *p* = 0.11). Unexpectedly, we also found that the main effect of RSA reactivity on internalizing symptoms was specific to anxiety (*β* = −0.04, *z* = −3.02, *p* = 0.002 for combined separation and overanxious symptoms; *β* = −0.01, *z* = −0.81, *p* = 0.42 for depression subscale). The two‐way interaction between RSA reactivity and positive affect predicting internalizing symptoms was trending toward significance for combined anxiety symptoms (*β* = −0.03, *z* = −1.79, *p* = 0.074) but was nonsignificant for depressive symptoms (*β* = −0.01, *z* = −0.74, *p* = 0.46). Of note, this overall pattern of specificity for anxiety symptoms could be because of the low base rate of depressive symptoms in the present sample, and all observed effects were larger when considering combined internalizing symptoms than anxiety alone.

## 4 Discussion

The aims of the current study were to (1) clarify associations between cardiovascular and behavioral measures of emotional reactivity across two tasks designed to elicit social fear and positive affect and (2) examine the unique and combined associations between cardiovascular and behavioral reactivity during these tasks and internalizing symptoms. Findings were consistent with emotion‐specific patterns of autonomic activation, such that behavioral displays of positive affect during the parent task were associated with RSA withdrawal, but no significant associations emerged between autonomic reactivity and behavioral displays of fear during the stranger task. Further, RSA augmentation during the dyadic positive affect task predicted lower internalizing symptoms. Behavioral displays of fear and positive affect were not associated with internalizing symptoms when considered as main effects. However, higher behavioral displays of stranger fear did predict higher internalizing symptoms when coupled with reciprocal parasympathetic activation. These findings suggest context‐specific patterns of autonomic activation that are differentially linked with internalizing symptoms and support the utility of cardiovascular markers of emotional reactivity in characterizing risk for internalizing psychopathology.

Some theoretical perspectives posit that fluctuations in RSA reflect undifferentiated emotional arousal (Brosschot et al. 2016; Brosschot et al. 2017; Porges 1995, 2007), whereas others suggest emotion‐specific patterns of autonomic activation (Fry et al. 2022; Levenson et al. 1990). Our findings were consistent with this latter interpretation, such that behavioral displays of positive affect during the parent task were associated with RSA withdrawal and no changes in PEP, but there were no significant associations between behavioral displays of fear during the stranger task and the autonomic measures. Recent studies have distinguished approach‐ and avoidance‐oriented emotions as having unique autonomic correlates to facilitate distinct behavioral goals (Fry et al. 2022; Gatzke‐Kopp and Ram 2018). Specifically, RSA withdrawal may be observed in the presence of an approach‐oriented behavioral response (e.g., displays of positive affect) but not in the context of freezing or avoidance (e.g., behavioral displays of fear). The literature shows inconsistent associations between fear and RSA, such that some studies report that higher fear is associated with RSA withdrawal (Quiñones‐Camacho and Davis 2019; Viana et al. 2021), whereas others report that greater fear is associated with RSA augmentation (Buss et al. 2018; Calkins and Dedmon 2000; Talge et al. 2008). Notably, studies linking RSA withdrawal to fearful behavior have used behavioral codes consistent with mobilization, such as running away or crying (Quiñones‐Camacho and Davis 2019), whereas links between RSA augmentation and fear are observed when immobilization or freezing is the predominant behavioral response (Buss et al. 2018). We were unable to parse mobilization versus freezing behaviors from the behavioral codes in this study, as the bodily fear and avoidance codes included both types of behaviors (e.g., startling, turning body away, covering face). As such, this heterogeneity in the association between RSA reactivity and fear, which may explain the absence of a main effect in this study, could be a result of variation in the kinds of fear‐related behaviors employed. However, this explanation remains to be empirically tested.

Consistent with expectations, we found that RSA reactivity during the positive affect task was negatively related to internalizing symptoms, such that RSA withdrawal in this context was associated with higher internalizing symptoms, and RSA augmentation was associated with lower internalizing symptoms. Critically, this task is designed to elicit positive affect during an interaction between the child and their caregiver. As such, RSA augmentation in this context aligns with polyvagal models of social affiliation (Porges 1995, 2007), which posit that parasympathetic augmentation promotes social engagement behaviors and feelings of safety and trust (Porges and Carter 2017). Therefore, RSA augmentation during this task may reflect a more positive parent–child attachment relationship, which is a well‐established predictor of positive socioemotional outcomes in children (Cooke et al. 2019; Hill et al. 2023; Ranson and Urichuk 2008). Consistent with this interpretation, the association between RSA reactivity and internalizing symptoms was only significant at average or high levels of positive affect, suggesting that RSA augmentation in this context may protect against internalizing difficulties by facilitating expressions of positive affect between the child and their caregiver. At the same time, we observed group‐level decreases in RSA from baseline to the positive affect task, which suggests that RSA withdrawal during this task is, to some extent, normative, and there may not be a single physiological pattern that is adaptive in this context. An alternative interpretation of these findings is that RSA augmentation during this task reflects a subgroup of children with exuberant temperament, which relates to a lower risk for internalizing but a higher risk for externalizing problems (Davies et al. 2024; McDoniel and Buss 2018; Morales et al. 2015). Indeed, other work has found a positive association between exuberance and RSA augmentation during the same positive affect task used in the current study (Morales et al. 2015). This interpretation is also consistent with the concurrent pattern of high positive affect and RSA augmentation predicting lower internalizing symptoms. More work is needed to test these competing predictions regarding the role of positive affect and RSA augmentation in shaping risk or resilience for psychopathology.

Finally, it is also important to note that high positive affect and concurrent RSA withdrawal were related to higher levels of internalizing symptoms. Notably, because the data are cross‐sectional, we cannot tease apart the directionality of reactivity and internalizing symptoms, and reactivity patterns during the task may be dependent on the child's level of internalizing problems. For example, children with higher levels of internalizing symptoms may expend more physiological resources (i.e., RSA withdrawal) to display positive affect. Future longitudinal work is needed to disentangle the temporal relation between positive emotional reactivity and internalizing symptoms.

Contrary to expectations, behavioral displays of positive affect during the parent task were positively related to internalizing symptoms. However, this association was attenuated when controlling for physiology. Many studies have reported an inverse relation between positive affect and internalizing (Hankin et al. 2017; Khazanov and Ruscio 2016; Morales et al. 2022; Watson et al. 2005). However, these studies often employ self‐ or parent‐reported measures of general positive affect or assess positive affect across several tasks, which may be more reliable indices of internalizing risk than observed positive affect within a single laboratory task. Future work should employ multiple measures of positive affect or positive valence system function (e.g., self‐report, behavior, neurophysiology) to assess their associations with each other and potentially unique associations with internalizing symptoms, particularly given mounting evidence that positive valence system function plays a central role in psychopathology risk (Hankin et al. 2017; Khazanov and Ruscio 2016). Alternatively, this pattern of findings could be due to the low level of depressive symptoms in the present sample, as positive affect is more strongly related to the development of depression than anxiety. Nevertheless, these findings do suggest that when considering physiological and behavioral markers of positive affect during a parent–child interaction, the physiological markers may be more relevant to predicting risk for internalizing symptoms. Other studies have also demonstrated the unique clinical utility of RSA in characterizing parent–child dynamics and psychopathology risk (T. P. Beauchaine 2015; Lunkenheimer et al. 2018).

Finally, we found no main effects of behavioral or autonomic reactivity during the stranger conversation task on internalizing symptoms, which was inconsistent with expectations. However, we did find an interaction between fearful behavior and autonomic reactivity in characterizing internalizing symptoms, such that greater fear coupled with a reciprocal parasympathetic response predicted higher internalizing symptoms. This finding is consistent with prior work on dysregulated fear, which demonstrated that higher task‐specific fear was associated with greater task RSA, especially for episodes characterized by social threat (Buss et al. 2018). Critically, the primary behavioral response for these children in fear‐eliciting contexts is to freeze (Buss 2011; Buss et al. 2018), which may be supported by reciprocal parasympathetic activation. Children who show this biobehavioral immobilization response in contexts where there is an expectation to engage, as in the current fear task, may be at higher risk for internalizing difficulties. Supporting this idea, other studies indicate that failure to withdraw parasympathetic resources in response to an environmental threat or challenge is associated with internalizing risk (Graziano and Derefinko 2013; Obradović and Finch 2017). When considering the combined effects of RSA and PEP, some studies suggest that uncoordinated activation may be the adaptive physiological response to avoidance‐oriented emotions such as fear, simultaneously facilitating vigilance and focused attention (Fry et al. 2022; Gatzke‐Kopp and Ram 2018). Taken together, these studies and our current findings suggest that fearful behavior alone, at least in a single task, may not be sufficient to predict which children are at risk for developing internalizing problems. Rather, patterns of physiological regulation may be important in distinguishing which fearful children are at risk.

### 4.1 Limitations and Future Directions

The present study has several strengths, including multimodal measurement of emotional reactivity, a focus on two distinct emotions relevant to internalizing symptom development, and the use of a middle childhood sample to characterize these associations before the high‐risk developmental period of adolescence and inform the study of subsequent trajectories. Our study's limitations could guide future work. First, our sample exhibited mostly subclinical levels of internalizing symptoms. Internalizing disorders in middle childhood likely involve differences in etiology from the emergence of subclinical symptoms, including differences in autonomic development (Dietrich et al. 2007; El‐Sheikh et al. 2013). As such, our findings can only be interpreted as relating to the emergence of subclinical symptoms, and the results may differ for children with diagnosed internalizing disorders. Relatedly, due to the cross‐sectional design, we cannot infer that these patterns of emotional reactivity precede the development of internalizing symptoms. However, by focusing on subclinical internalizing symptoms in middle childhood, we were able to identify patterns of emotional reactivity that characterize the early emergence of symptoms, one of the best predictors of later disorder (Keenan et al. 2009; Steinsbekk et al. 2022). Further, these patterns lay the groundwork for understanding future change across adolescence in ways that potentially confer risk for internalizing disorders. Future work should examine whether these patterns of emotional reactivity prospectively predict internalizing symptoms or change across development, particularly across the peripubertal period when substantial neurobiological and social changes are underway (Nelson et al. 2016; Pfeifer and Allen 2021).

Third, we focused on positive affect and fear in the present study because of their robust associations with internalizing symptomatology (Fox and Pine 2012; Hettema et al. 2020; Klein and Mumper 2018; Kujawa et al. 2020; Morales et al. 2022). Sadness‐inducing contexts are also relevant to internalizing symptom development and should be considered in future studies.

Fourth, we used mean‐level scores of the emotional reactivity measures rather than examining within‐person dynamics. The latter may reveal more nuanced associations between autonomic and behavioral reactivity that better characterize internalizing risk, especially given potentially competing functions of the autonomic nervous system within these tasks. Specifically, the positive affect task may require both RSA withdrawal to facilitate behavioral displays of positive affect (Fry et al. 2022; Porges 2007, 2009) and RSA augmentation to facilitate feelings of social affiliation (Porges and Carter 2017), whereas the fear task may require different autonomic processes to facilitate both vigilance and focused attention (Fry et al. 2022; Gatzke‐Kopp and Ram 2018). Relatedly, we used 30‐s epochs to calculate RSA and PEP, which is a common approach in the literature on emotional reactivity (e.g., Andersen et al. 2020; Fortunato et al. 2013; Lunkenheimer et al. 2018). However, this yielded a low number of epochs, particularly for the positive affect task, and future work to replicate these findings using dynamic measures of autonomic reactivity (e.g., moving window averages) and to demonstrate sufficient test–retest reliability of physiological reactivity measures is warranted.

Finally, our sample was predominantly White and middle‐class. While a shy or fearful temperament, as assessed during the stranger conversation task, is consistently associated with poorer adjustment and socioemotional well‐being in Western cultures (Buss et al. 2013; Goldsmith et al. 2022; Rubin et al. 2009), the opposite pattern may be observed in cultures that value group‐level harmony over individual‐level assertiveness or social initiative (Chen et al. 1998; Chen 2019). Therefore, patterns of physiological and behavioral responses and their relation to internalizing symptoms, particularly for fear‐eliciting tasks, may differ for children from Eastern cultures who are raised with different socialization goals (Chen 2019).

## 5 Conclusion

The current study augments the existing literature by examining the unique and combined associations between autonomic and behavioral indices of emotional reactivity and internalizing symptoms, focusing on two temperamental traits central to etiological models of internalizing risk. Behavioral displays of positive affect with a parent were associated with RSA withdrawal but no sympathetic change, and we did not find associations between behavioral displays of stranger fear and autonomic reactivity. RSA augmentation during a dyadic positive affect task with a parent was associated with lower internalizing symptoms, whereas reciprocal parasympathetic activation coupled with higher behavioral displays of stranger fear was associated with higher internalizing symptoms. The results highlight the utility of multimodal assessment of emotional reactivity across physiological and behavioral levels and lay the groundwork for future work to delineate if and how these patterns of emotional reactivity change across adolescence in ways that potentially confer risk for internalizing disorders.

## Ethics Statement

The Institutional Review Board at the University of Wisconsin–Madison approved this study. Parents of children provided informed consent, and all children provided informed assent

## Conflicts of Interest

The authors declare no conflicts of interest.

## Supporting information



Descriptive statistics and bivariate correlations between individual behaviors during the dyadic positive affect task (Table S1) and stranger fear task (Table S2) are provided in Supporting Information.

## Endnotes

1 The significance and direction of all reported effects remained consistent when controlling for the length of the stranger task.

2 To understand the specificity of effects, we also examined externalizing symptoms as an outcome. No main effects or interactions were significant (*p*s > 0.05).
