# Supplementary material for: Emotional Reactivity and Internalizing Symptoms in Middle Childhood: Integrating Autonomic and Behavioral Markers of Social Fear and Positive Affect
Source: Dev Psychobiol. 2025 Jun 17;67(4):e70056. doi: 10.1002/dev.70056 (PMC12171948; doi:10.1002/dev.70056)
Supplement: Supplementary file 1 — Descriptive statistics and bivariate correlations between individual behaviors during the dyadic positive affect task (Table S1) and stranger fear task (Table S2) are provided in Supporting Information. [file DEV-67-e70056-s001.docx]

Table S1

*Descriptive statistics and bivariate correlations between individual behaviors during the dyadic positive affect task*

| Variable | *M* (*SD*) | Min. | Max. | 1 | 2 | 3 |
| --- | --- | --- | --- | --- | --- | --- |
|  |  |  |  |  |  |  |
| 1. Intensity of smiling | 2.05 (.53) | .44 | 3.00 |  |  |  |
|  |  |  |  |  |  |  |
| 2. Enthusiasm | 2.14 (.40) | .22 | 2.89 | .37** |  |  |
| 3. Intensity of laughter | .26 (.21) | .00 | 1.00 | .43** | .24** |  |
|  |  |  |  |  |  |  |
| 4. Positive vocalizations | .21 (.30) | .00 | 2.00 | .37** | .35** | .37** |
|  |  |  |  |  |  |  |

*Note.* * indicates *p* < .05, ** indicates *p* < .01 (1-tailed).

Table S2

*Descriptive statistics and bivariate correlations between individual behaviors during the stranger fear task*

| Variable | *M* (*SD*) | Min. | Max. | 1 | 2 | 3 | 4 |
| --- | --- | --- | --- | --- | --- | --- | --- |
|  |  |  |  |  |  |  |  |
| 1. Avoidance | .14 (.30) | .00 | 2.00 |  |  |  |  |
|  |  |  |  |  |  |  |  |
| 2. Bodily fear | .29 (.56) | .00 | 2.70 | .13* |  |  |  |
|  |  |  |  |  |  |  |  |
| 3. Negativity | .23 (.32) | .00 | 1.00 | .62** | .65** | .18** |  |
| 4. Latency to first fear response (*s*) | 227.83 (210.12) | 1 | 445 | -.37** | -.48** | -.16** | -.65** |

*Note.* * indicates *p* < .05, ** indicates *p* < .01 (1-tailed).
